# Assessing Carbon Monoxide Exposure in Food Delivery Workers by Using Exhaled Air with Consideration of Smoking Habits

**DOI:** 10.1007/s44197-025-00468-w

**Published:** 2025-10-20

**Authors:** Kun-Hua Li, Ya-Yun Cheng, Shih-Chieh Huang, Chi-An Chen, Jhih-Yuan Lu, Zih-Ting Chao, Yen-Cheng Tseng, How-Ran Guo

**Affiliations:** 1 Department of Environmental and Occupational Health, College of Medicine, National Cheng Kung University, Tainan, Taiwan; 2 School of Medicine, College of Medicine, National Sun Yat-Sen University, Kaohsiung, Taiwan; 3 Department of Tourism, Food & Beverage Management, Chang Jung Christian University, Tainan, Taiwan; 4 Department of Occupational and Environmental Medicine, National Cheng Kung University Hospital, Tainan, Taiwan; 5 Research Center of Occupational Safety, Health, and Medicine, National Cheng Kung University, Tainan, Taiwan

**Keywords:** Carbon monoxide, Exhaled air, Food delivery worker, Online food delivery service, Occupational exposure, Traffic emission

## Abstract

**Background:**

The number of food delivery workers engaging in online food delivery (OFD) services has soared recently. While they are exposed to air pollutants from traffic exhaust frequently, their exposure to carbon monoxide (CO) is seldom assessed. This study aimed to assess the CO exposure of food delivery workers using exhaled air with consideration of smoking habits.

**Methods:**

We recruited food delivery workers from OFD services and compared their CO concentrations in exhaled air with those of a reference group. In addition, we used a questionnaire to gather data on sociodemographic characteristics, health behaviors, health status, and work-related conditions.

**Results:**

We enrolled 156 food delivery workers and a reference group of 49 members. The results showed that food delivery workers had a higher mean CO concentration in exhaled air (4.79 ppm vs. 1.51 ppm, *p* < 0.001) as well as a higher proportion of smokers (25.0% vs. 4.1%, *p* < 0.001). While food delivery workers with smoking habits had a much higher mean CO concentration in exhaled air than those without smoking habits (14.46 ppm vs. 1.57 ppm, *p* < 0.001), amongst non-smokers, the CO concentration was still higher in food delivery workers than in the reference group (1.57 ppm vs. 1.13 ppm, *p* = 0.002). Among smokers, smoking might contribute 89% of the CO in exhaled air.

**Conclusions:**

Food delivery workers had higher CO concentrations in exhaled air than the reference group, indicating higher exposure levels. Smoking is an important contributing factor when using exhaled air for CO exposure assessment.

## Background

Carbon monoxide (CO) is a common pollutant produced by the incomplete combustion of carbon-based fuels in gasoline and diesel engines, known for its affinity for hemoglobin, which is approximately 200 times greater than that of oxygen. This can lead to remarkable tissue hypoxia in organs and systems during acute exposure, known as CO poisoning. CO poisoning is associated with a wide range of acute symptoms, including hypotension, ventricular arrhythmia, headache, and drowsiness, which can be life threatening [1, 2]. Beyond acute intoxication, chronic low-level exposure to CO may lead to adverse health effects through various potential mechanisms, such as oxidative stress, systemic inflammation, and vascular endothelial injury [3–6]. Epidemiological studies have associated ambient CO exposure with the onset or exacerbation of various conditions, including neurodegenerative [7–9], cardiovascular [10–15], cerebrovascular [16], respiratory [10, 17–19], and renal diseases [20, 21], warranting further exploration of occupations at risk.

Epidemiological research has consistently shown that occupational groups affected by motor vehicle exhaust, such as traffic police officers and street vendors, have elevated levels of carboxyhemoglobin (COHb) in their blood and greater personal environmental exposure relative to other occupational groups [22–24]. However, invasive procedures like blood collection add to the complexity and concerns of conducting epidemiological surveys. On the other hand, the measurement of CO concentrations in exhaled air is a well-established non-invasive method, widely used in clinical settings for various purposes, including smoking cessation programs, pregnancy guidance, health education, and clinical investigations [25]. Additionally, it has been demonstrated that this testing modality can serve as a valid measure of short-term exposure to ambient CO pollution [26]. In addition to its non-invasive nature, which may reduce anxiety among participants and increase their willingness to participate, it is cost-effective. Furthermore, the devices used can provide immediate monitoring data, which adds to the convenience of data collection and may be used for preventing further excess exposure in high-risk populations. The influence of the smoking habit is a limitation of this biomarker for assessing CO exposure, but few studies have been conducted to evaluate the magnitude of the effect.

The use of online food delivery (OFD) services has soared in recent years, attributable to the convenience they offer and changing consumer behaviors in modern society [27]. The COVID-19 pandemic further accelerated the growth of OFD services, spurred by social distancing policies implemented by many countries, along with increased global awareness and concern regarding infectious diseases [28]. These services have provided part-time and full-time employment opportunities for numerous food delivery workers. Notably, these workers are routinely exposed to traffic-related air pollution due to the nature of their job. However, research into their occupational exposure to CO remains limited.

Taiwan is notable for its high density of motorcycles, particularly motor scooters, which surpasses that of most other countries globally [29]. This prevalence is attributable to Taiwan’s unique cultural, geographical, and economic characteristics, making motor vehicle exhaust one of important contributors to ambient air pollution on the island. Motor vehicle exhaust consist of a variety of gaseous pollutants, including CO, and so workers who deliver food by riding motorcycles are at high risk of exposure to CO. Therefore, this study aimed to estimate the occupational CO exposure in food delivery workers using exhaled air and to evaluate the effect of smoking habits on CO in exhaled air.

## Materials and Methods

### Study Design and Study Sample

The current survey assessed occupational CO exposure by measuring its concentration in exhaled air and collected data using a self-reported questionnaire. We recruited participants through posters, leaflets, and social media platforms in 2021 in Tainan, Taiwan. Eligible participants were at least 20 years old and could communicate in Mandarin or Taiwanese. Food delivery workers, either part-time or full-time employees of OFD services, were required to provide proof of employment by displaying relevant smartphone application(s). Healthcare workers and college students were recruited as the reference group. Individuals with notifiable respiratory infectious diseases, such as tuberculosis and pertussis, were excluded for safety reasons. Additionally, those presenting with symptoms of upper respiratory infections, including fever, were excluded from the survey.

### Sample Size Estimation

The sample size for the present study was estimated using G*Power 3.1 (Heinrich Heine University Düsseldorf, Düsseldorf, Germany) to ensure adequate statistical power [30]. To address the potential for a skewed distribution of the exposure measurements, as indicated in a previous study [24], we determined the sample size using the Wilcoxon-Mann-Whitney method with a two-tailed test design in the software. The type I (alpha) error level was set at 0.05, the statistical power at 0.9, and the effect size at 1.47, which was based on the results of an occupational CO exposure assessment study related to traffic exhaust [23]. The calculations indicated that at least 12 participants in each group were needed to achieve sufficient statistical power to detect the effect between the two study groups.

### Questionnaire

A questionnaire for in-person interviewing was used to collect data on smoking habits as well as sociodemographic characteristics (age, sex, educational qualifications), working conditions (tenure in previous and current positions, average working hours per day), environmental exposure conditions (exposure to cooking fumes, traffic exhaust, and industrial odors in the living place), transportation (primary modes of transportation, average usage hours per day), other health behaviors (living with smokers, regular exercise practices), health status, and medical history, including lactose intolerance and irritable bowel syndrome. Five well-trained team members administered the questionnaire.

The questionnaire had undergone an expert validity assessment and a test–retest reliability assessment at a 4-week interval. The kappa coefficients ranged from 0.80 to 1.00, weighted kappa coefficients from 0.62 to 1.00, and intra-class correlation coefficients from 0.72 to 0.89, with all 95% confidence intervals (CIs) not spanning zero.

### The Concentration of CO in Exhaled Air

We used the Micro^+^ Smokerlyzer (Bedfont Scientific Ltd, Maidstone, England) to measure the concentrations of CO (in ppm) in exhaled air. This device can also simultaneously provide the percentage of COHb (%COHb), which was calculated using regression equations [25]. Its measurement validity has been established through academic research comparing the CO concentrations in exhaled air with COHb levels in blood [31]. The devices used in the current study were calibrated before the survey commenced. According to the manufacture’s instruction, we measured each participant’s CO concentrations in exhaled air twice, 5 min apart, and the mean of these two concentrations was calculated as the final reading.

### Statistical Analysis

Differences between food delivery workers and the reference group were evaluated using the chi-square test or Fisher’s exact test with zero-count adjustment for categorical variables, independent t test for continuous variables, and the Wilcoxon rank-sum test for ordinal variables or non-normally distributed continuous variables. As a sensitivity analysis, participants who reported having lactose intolerance or irritable bowel syndrome were excluded, because these conditions could generate additional hydrogen in the gastrointestinal tract, potentially altering the CO concentration measurements in exhaled air. All analyses were conducted using R version 4.1.2 (R Project for Statistical Computing, Vienna, Austria), with a two-tailed test and a type I error level of 0.05.

## Results

The present study recruited 205 participants, including 156 food delivery workers, along with 16 healthcare workers and 33 college students comprising the reference group. The food delivery workers were more likely to be male (69.9% vs. 24.5%, *p* < 0.001) and older (mean age, 34.2 years vs. 26.3 years, *p* < 0.001) compared with the reference group (Table 1). There were more current smokers among food delivery workers (25.0% vs. 4.1%, *p* < 0.001), but the proportion of members living with smokers was similar between the two groups. The two groups had similar daily mean working hours, but food delivery workers had shorter tenures in the current job (mean, 1.3 years vs. 3.4 years, *p* < 0.001). Regarding self-perceived exposure at the residence, while the two groups reported similar exposures to traffic exhaust and industrial odors, a higher proportion of food delivery workers reported daily exposure to cooking fumes (12.8% vs. 0.0%, *p* < 0.001). All food delivery workers used motor scooters as their primary mode of transportation (100.0% vs. 93.9%, *p* = 0.013), and they reported longer mean daily usage (8.5 h vs. 1.1 h, *p* < 0.001). A lower proportion of food delivery workers reported regular exercise (38.5% vs. 55.1%, *p* = 0.059), but the difference did not reach statistical significance. Self-reported health status and prevalence of lactose intolerance and irritable bowel syndrome were comparable between the two groups.

**Table 1:** Characteristics and results of the questionnaire survey between the study groups

	Food delivery workers (*n* = 156)		Reference group (*n* = 49)		*P*-value
	Frequency (%)	Mean ± SD	Frequency (%)	Mean ± SD	
Characteristics					
Sex^a^					< 0.001
Male	109 (69.9)		12 (24.5)		
Female	47 (30.1)		37 (75.5)		
Age (year)^b^		34.2 ± 8.3		26.3 ± 6.3	< 0.001
Educational qualifications^c^					0.082
High school and below	74 (47.4)		16 (32.7)		
Undergraduate	79 (50.6)		30 (61.2)		
Graduate	3 (2.0)		3 (6.1)		
Working conditions					
Tenure (year)^c^		1.3 ± 1.0		3.4 ± 3.6	< 0.001
Working hours (per day)^b^		7.8 ± 3.0		7.4 ± 3.3	0.483
Environmental exposure conditions					
Perceiving cooking fume in living place^a, d^					0.006
No	113 (72.5)		34 (69.4)		
Not everyday	23 (14.7)		15 (30.6)		
Everyday	20 (12.8)		0 (0.0)		
Perceiving traffic exhaust in living place^a^					0.471
No	130 (83.3)		43 (87.8)		
Not everyday	7 (4.5)		3 (6.1)		
Everyday	19 (12.2)		3 (6.1)		
Perceiving industrial odors in living place^a, d^					0.764
No	149 (95.5)		47 (95.9)		
Not everyday	4 (2.6)		2 (4.1)		
Everyday	3 (1.9)		0 (0.0)		
Transportation					
Primary mode of transportation^a, d^					0.013
Motorcycle	156 (100.0)		46 (93.9)		
Bicycle or walking	0 (0.0)		3 (6.1)		
Usage hours (per day)^b^		8.5 ± 3.2		1.1 ± 1.1	< 0.001
Health behaviors					
Smoking^a, d^					< 0.001
Current smoker	39 (25.0)		2 (4.1)		
Ex-smoker	8 (5.1)		0 (0.0)		
Non-smoker	109 (69.9)		47 (95.9)		
Living with smokers^a^					0.362
No	118 (76.1)		41 (83.7)		
Yes	37 (23.9)		8 (16.3)		
Exercising regularly^a^					0.059
No	96 (61.5)		22 (44.9)		
Yes	60 (38.5)		27 (55.1)		
Health status					
Self-perceived health status^c^					0.657
Extremely poor	4 (2.6)		0 (0.0)		
Poor	21 (13.5)		7 (14.3)		
Medium	72 (46.1)		22 (44.9)		
Good	50 (32.0)		17 (34.7)		
Excellent	9 (5.8)		3 (6.1)		
Medical history					
Lactose intolerance^a^					0.454
No	136 (87.2)		45 (91.8)		
Yes	20 (12.8)		4 (8.2)		
Irritable bowel syndrome^a^					0.416
No	139 (89.1)		46 (93.9)		
Yes	17 (10.9)		3 (6.1)		

SD: standard deviation. ^a^chi-squared test or Fisher exact test; independent t test; Wilcoxon rank-sum test; ^d^zero count adjustment: we added a small adjustment value of 0.5 in each cell

Food delivery workers had a higher mean CO concentration in exhaled air than that of the reference group (mean difference = 3.28 ppm; 95% CI: 1.83—4.73) (Table 2). Food delivery workers who were current smokers had a higher mean CO concentration than their smoking referents, but the difference did not reach statistical significance (14.46 ppm vs. 10.25 ppm; *p* = 0.660). Nonetheless, amongst food delivery workers, those with a smoking habit had a higher mean CO concentration than those without a smoking habit (14.46 ppm vs. 1.57 ppm; *p* < 0.001).

**Table 2:** The difference in exhaled CO concentration and percentage of carboxyhemoglobin between the study groups

	Food delivery workers	Reference group	Difference (95% confidence interval)
	Mean ± Standard deviation		
All participants	(*n* = 156)	(*n* = 49)	
Exhaled CO concentration (ppm)	4.79 ± 8.05	1.51 ± 2.48	3.28 (1.83, 4.73)
%COHb	1.36 ± 1.31	0.73 ± 0.52	0.63 (0.37, 0.87)
Excluding current smokers	(*n* = 117)	(*n* = 47)	
Exhaled CO concentration (ppm)	1.57 ± 0.76	1.13 ± 0.82	0.44 (0.15, 0.71)
%COHb	0.84 ± 0.22	0.67 ± 0.35	0.17 (0.03, 0.27)

After excluding smokers, who typically have higher CO concentrations in their exhaled air relative to non-smokers [32, 33], the difference between food delivery workers and the referents was smaller but still reached statistical significance (mean difference = 0.44 ppm; 95% CI: 0.15—0.71; Table 2). The transformed %COHb levels mirrored the findings observed with CO concentrations in exhaled air.

After excluding participants with self-reported lactose intolerance or irritable bowel syndrome, we found that food delivery workers still had a higher mean CO concentration in exhaled air than the reference group (mean difference = 3.83 ppm; 95% CI: 2.30—5.37) (Table 3) in the sensitivity analysis. When participants who were current smokers were further removed from the analysis, food delivery workers still had a higher mean CO concentration in exhaled air (mean difference = 0.33 ppm; 95% CI: 0.03—0.63; Table 3), consistent with the primary findings in Table 2. The transformed %COHb levels paralleled the results of the CO concentrations in exhaled air.

**Table 3:** Sensitivity analysis^a^ on the difference in exhaled CO concentration and percentage of carboxyhemoglobin between the study groups

	Food delivery workers	Reference group	Difference (95% confidence interval)
	Mean ± Standard deviation		
All participants	(*n* = 126)	(*n* = 43)	
Exhaled CO concentration (ppm)	4.98 ± 8.59	1.15 ± 0.83	3.83 (2.30, 5.37)
%COHb	1.38 ± 1.40	0.67 ± 0.35	0.71 (0.44, 0.98)
Excluding current smokers	(*n* = 95)	(*n* = 43)	
Exhaled CO concentration (ppm)	1.48 ± 0.77	1.15 ± 0.83	0.33 (0.03, 0.63)
%COHb	0.82 ± 0.24	0.67 ± 0.35	0.14 (0.02, 0.26)

^a^Excluding participants who self-reported suffering from lactose intolerance or irritable bowel syndrome

## Discussion

Food delivery workers in OFD services represent a rapidly emerging occupation globally, likely due to the flexible work arrangements compared with many other occupations. However, the swift growth of this workforce has raised several safety concerns, including the risk of overwork, dangerous driving behaviors, and exposure to traffic-related emissions and cooking fumes, which call for the attention of governments and researchers. Our study found that many food delivery workers with shorter tenures had joined the OFD sector during the COVID-19 pandemic. This probably indicate that their original employment or financial status was impacted by the social distancing policy and public concerns about the pandemic. Nonetheless, the attractive part-time income offered by OFD services may have encouraged workers to switch jobs or take on this job part-time even after the pandemic.

Unsurprisingly, all food delivery workers in our study used motor scooters as their primary mode of transportation. Motor scooters offer benefits, such as increased mobility, lower maintenance costs, and fewer parking challenges compared with other vehicles. Currently, approximately 14 million motorcycles, including motor scooters, are registered in Taiwan, nearly half the total population of the island, making motor vehicle exhaust one of the major contributors to ambient air pollution. Food delivery workers are one of the high exposure occupation groups of the pollutants.

Our findings revealed that food delivery workers had higher CO concentrations in exhaled air than that of the reference group, even after excluding smokers, who constituted a higher proportion of the members in the food delivery worker group than in the reference group (25.0% vs. 4.1%). However, after taking smoking into account, food delivery workers still had higher CO concentrations in exhaled air than the reference group, and so the potential bias would not change the conclusion that food delivery workers had higher CO exposure. Nonetheless, the finding that a higher proportion of current smoker were reported among food delivery workers indicates the potential benefit of introducing smoking cessation programs to this occupation group.

There were some limitations of this study that warrant discussion. Firstly, the food delivery workers recruited from the OFD services were all operating within a metropolitan area of southern Taiwan. Consequently, their occupational exposure to traffic-related CO pollution might be higher than their counterparts in rural areas. However, importantly, food delivery workers of OFD services tend to work in urban areas due to the consideration of the consumer density likely to use the service. Therefore, the real-world data collected in our study can provide an estimate that closely represents the typical occupational CO exposure for OFD workers in the study area. Secondly, the study relied on a self-reported questionnaire to collect data on major variables, such as current job, daily working hours, and health behaviors. It is possible that participants overstated or downplayed their actual circumstances, which might lead to social desirability bias, meaning that they might have responded in ways they perceived as more socially acceptable. However, the misclassification is likely to be random, if existing, because the social desirability bias applies to both study groups. Thirdly, we recruited voluntary participants through open recruitment instead of employing random selection from the target population. As a result, there were more food delivery workers than referents in the final study sample. Because we enrolled the participants by the order of registration, which applied to both groups, this is unlikely to introduce remarkable bias to the study results. The recruitment also led to differences between food delivery workers and the reference group in the distributions of some important factors such as smoking and sex, which might introduce bias to the study results. To address this issue, we had performed a sensitivity analysis which excluded smokers and found that food delivery workers still had higher CO concentrations in exhaled air. As to sex, when we performed separated analyses by sex, the difference reached statistical significance in males (2.42 ppm, *p* = 0.03), but not in females (2.12 ppm, *p* = 0.18), most likely due to the lack of statistical power because the difference was similar to that in males. Fourthly, while the sample size in the current study had provided enough statistical power to detect the difference in CO exposure between food delivery workers and the reference group even after exclusion of smokers and those with self-reported lactose intolerance or irritable bowel syndrome, it is insufficient for conducting multiple regression analysis to adjust for more than two potential covariates at the same time. Further studies with more participants are needed to confirm our findings.

While OFD service is a fast-growing industry, few, if any, studies have been reported in the literature regarding the workers’ exposure to air pollution, although it is quite evident. In most previous studies on occupation exposure to ambient air pollutants, environmental measurement data were used. On the other hand, those used personal biomarkers usually gathered the data through measuring samples obtained by invasive approaches. In the current study, we used a biological marker at the individual level that can be obtained through a non-invasive method. Nonetheless, our study showed that smoking is an important contributing factor when using exhaled air for CO exposure assessment, and its contribution appeared to be larger than occupational exposure in this study. These findings are compatible with the observations in a cross-sectional study of firefighters which found that cigarette smoking was a main contributing factor of CO concentrations in exhaled air [34]. Specifically, in both food delivery workers (1.57 ppm vs. 14.46 ppm; *p* < 0.001) and the reference group (1.13 ppm vs. 10.25 ppm; *p* < 0.001), the mean CO concentration in the exhaled air among non-smokers was only about 11% of that in smokers. While this implicates the importance of smoking cessation, it calls for caution in using this biomarker for environmental or occupational exposure assessment.

## Conclusions

Food delivery workers exhibited higher CO concentrations in exhaled air than that of the reference group after taking smoking into account, indicating a higher exposure level associated with their work. The findings in the current study support the use of CO concentration in exhaled air, a non-invasive method with relatively lower costs, as an indicator of occupational CO exposure. Additionally, we identified smoking as an important source of CO exposure which contributed about 89% of the CO concentrations in exhaled air among smokers. This indicates a potential benefit of implementing smoking cessation programs and calls for caution in using CO concentrations in exhaled air for environmental or occupational exposure assessment.

## Data Availability

The datasets used and analyzed during the current study are available from the corresponding author on reasonable request.

## Abbreviations

CO: Carbon monoxide
COHb: carboxyhemoglobin
COVID-19: coronavirus disease 2019
CI: confidence interval
OFD: online food delivery
OR: odds ratio
